# High metabolic activity in positron emission tomography and systemic inflammation occurring years after exposure cessation in engineered stone silicosis

**DOI:** 10.1038/s41598-025-10562-5

**Published:** 2025-07-14

**Authors:** Antonio León-Jiménez, Julio Rodríguez-Rubio Corona, Gema Jiménez-Gómez, María Luisa Piñero Fernández-Reyes, Antonio Hidalgo-Molina, Magdalena Pajares-Vinardel, Miguel Ángel Conde-Sánchez, Antonio Campos-Caro, Daniel Sanchez-Morillo

**Affiliations:** 1 Pneumology Department, Puerta del Mar University Hospital, 11009 Cádiz, Spain; 2 Biomedical Research and Innovation Institute of Cadiz (INiBICA), 11009 Cádiz, Spain; 3 Nuclear Medicine Department, Puerta del Mar University Hospital, 11009 Cádiz, Spain; 4 Radiology Department, Puerto Real University Hospital, Puerto Real, 11510 Cádiz, Spain; 5 Genetics Area, Biomedicine, Biotechnology and Public Health Department, School of Medicine, University of Cádiz, 11510 Cádiz, Spain; 6 Department of Engineering on Automation, Electronics and Computer Architecture and Networks, University of Cádiz, 11510 Cádiz, Spain; 7 Área Genética. Departamento Biotecnología y Salud Pública, Universidad de Cádiz, 11510 Cádiz, Spain

**Keywords:** Silicosis, Engineered stone, Systemic inflammatory indices, Lymphocyte subsets, Positron emission tomography, Predictive markers, Prognostic markers, Respiratory tract diseases

## Abstract

Engineered stone silicosis is an interstitial lung disease that progresses rapidly; in many cases, it can cause respiratory insufficiency and death. The metabolic activities occurring in the lungs and adenopathies, as well as their relationships with systemic inflammation, are unknown. Patients with complicated silicosis were enrolled in this study. All of the patients had worked for at least 5 years in finishing and installing engineered stone and had not been exposed to these working conditions for at least 7 years. Clinical data measurements, positron emission tomography/computed tomography using ^18^F-fluorodeoxyglucose (^18^F-FDG PET/CT), respiratory function tests and blood samples were performed. The mean age of the patients was 44 ± 5.4 years. Moreover, the average exposure duration was 10.94 ± 3.2 years, and the average number of years from cessation of exposure was 11.6 ± 1.6 years. The average maximum standardized uptake value (SUVmax) of large opacities was 6.32 ± 3. All of the patients demonstrated hypermetabolic mediastinal lymphadenopathies, and 88.2% of the patients also demonstrated extrathoracic lymphadenopathies. The SUVmax of the large opacities was correlated with fibrinogen (*ρ *= 0.717, *P* = 0.001), the lymphocyte-to-monocyte ratio (*ρ *= − 0.506, *P* = 0.038), the systemic inflammatory response index (*ρ *= 0.559, *P* = 0.02) and CD4^+^NKT cells. Large areas of lung opacity and lymphadenopathies exhibited high metabolic activities years after the cessation of silica exposure. The relationships between metabolic activity and several inflammatory factors may lead to the exploration of new therapeutic targets.

**Supplementary Information:**

The online version contains supplementary material available at 10.1038/s41598-025-10562-5.

Engineered stone silicosis is a serious occupational health issue, with a high number of cases being reported in many countries, such as Australia, Spain, Israel and the United States, among others^1–3^; this high number of cases is attributed to the emergence of new materials being used in bathrooms and kitchen countertops. Moreover, this high incidence has even resulted in the prohibition of this material in Australia and proposals for its gradual ban in other European countries^4^. This type of engineered stone (ES), which is commonly known as artificial stone, quartz or silica agglomerates, is characterized by a high content of micronized crystalline silica (> 80% quartz and/or cristobalite), along with resins and metals^5,6^. Compared with natural stones, artificial silica agglomerates induce a more aggressive form of silicosis with a higher mortality rate^7^, and the disease can progress rapidly even after the cessation of exposure^8^.

Silicosis is a progressive interstitial lung disease with no specific treatment (except for lung transplantation, which is performed in the final stages of the disease). Despite the performance of numerous studies (most of which have been conducted in animal models and cell cultures)^9^, the underlying mechanisms and cellular processes involved in disease progression remain largely unknown.

Due to the fact that activated inflammatory cells consume glucose for energy and increase the expression of glucose transporters^10^, the use of positron emission tomography/computed tomography (PET/CT) with ^18^F-fluorodeoxyglucose (^18^F-FDG) may provide new insights into the roles of biomarkers and inflammatory cells that are involved in the progression of silicosis in afflicted patients.

Therefore, the aim of our study was to assess the metabolic activities of lung lesions and lymph nodes in patients with complicated silicosis due to ES, as well as to investigate their relationships with specific biomarkers, systemic inflammatory indices, and lymphocyte subpopulations in the peripheral blood.

## Results

### Silicotic conglomerates and metabolic activity

**Fig. 1:**
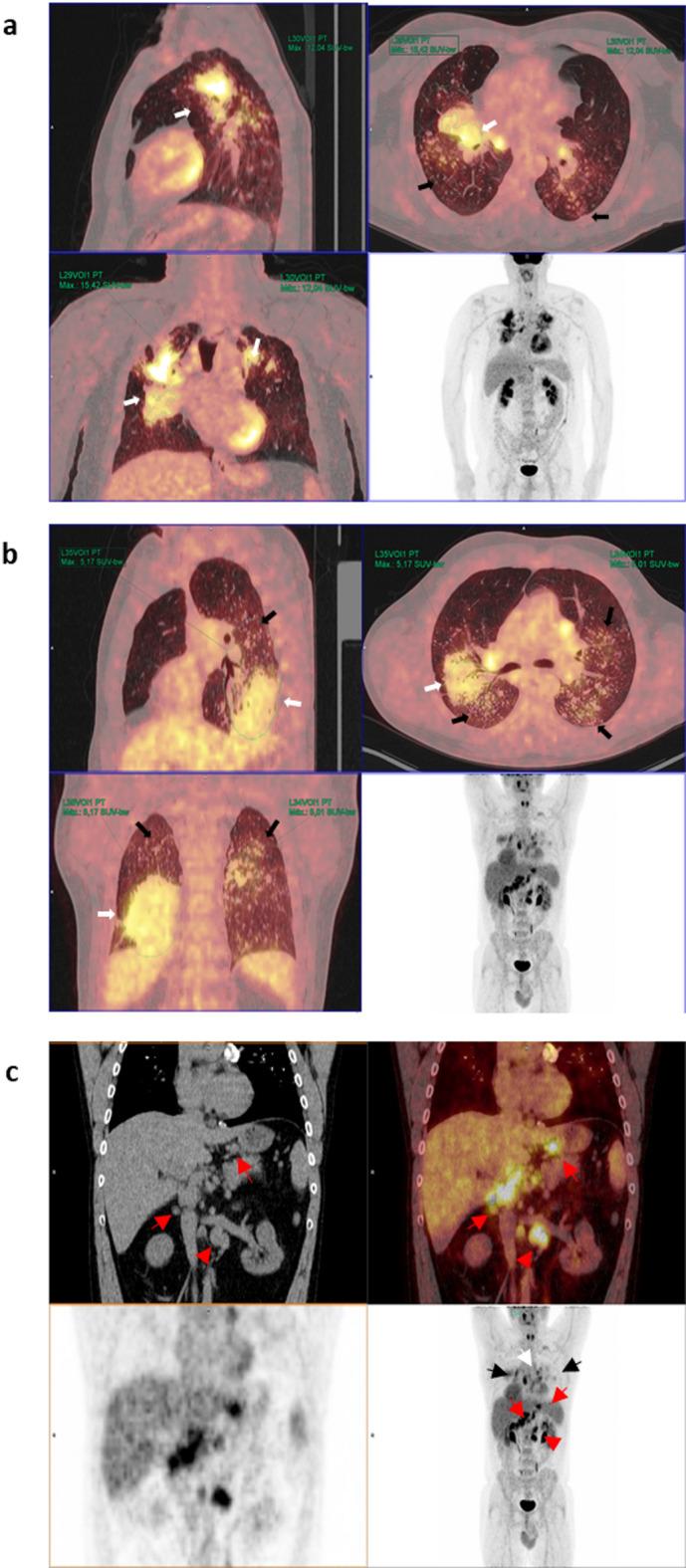
Representative PET/CT images from patients with: (**a**) high and (**b**) low SUVmax values and (**c**) hypermetabolic lymphadenopathy in other extra thoracic areas. (**a**) Maximum intensity projection (MIP) PET images (bottom right) and sagittal, axial and coronal and PET/CT images of a patient with bilateral pulmonary heterogeneous condensations (white arrows) showing intense metabolic activity up to an SUVmax of 15.42 in the right upper lobe, as well as scattered millimetric nodules (black arrows). (**b**) MIP PET images and in the sagittal, axial and coronal PET/CT images showing bilateral pulmonary condensations, which are larger on the right side (white arrows), and numerous bilateral millimeter-sized nodules (black arrows). Slight diffuse and heterogeneous increased uptake (up to an SUVmax of 5.17) is observed, even in areas of apparently healthy lung parenchyma. (**c**) PET, CT and PET/CT images in coronal and MIP sections in which, in addition to bilateral pulmonary (black arrows) and mediastinal (white arrow) involvement, multiple abdominal lymphadenopathies are observed in the celiac space, gastrohepatic ligament and retroperitoneum (red arrows), even below the renal artery (red arrowhead).

Radiological evaluation by high-resolution computed tomography (HRCT) was utilized to classify patients according to the International Classification of HRCT for Occupational and Environmental Respiratory Diseases (ICOERD) classification guidelines, with five patients being categorized as progressive massive fibrosis (PMF) category A, six as category B, and six as category C. All of the patients presented with bilateral large opacities (except for one patient who had a unilateral large opacity). The silicotic conglomerates (large opacities) exhibiting the highest uptakes were mostly located in the upper lobes. From each lung, the region with the highest SUVmax was selected. Representative images from patients with high and low SUVmax values are shown in Fig. 1. The average SUVmax of these pulmonary opacities was 6.3 ± 3.0 (range: 2.2–13.7). The values for each patient and the diameter of each opacity, which was measured in millimeters (mm) via high-resolution computed tomography (HRCT), are shown in Table 1.

**Table 1:** Relationships between radiological opacities in the lung and their corresponding metabolic activity measured by the suvmax.

Patient	ILO/ICOERD category	Diameter right lung opacity	Diameter left lung opacity	Average diameter pulmonary opacities	Location	SUVmax right lung opacity	SUVmax Left lung opacity	Average SUVmax pulmonary opacities
1	B/B	41	28	34	RUL-LUL	6.0	7.7	6.9
2	B/B	37	25	31	RUL-LUL	8.5	10.7	9.9
3	C/C	60	24	42	RLL-LLL	5.1	5.0	5.0
4	B/C	51	36	43.5	RLL-LLL	9.4	8.8	9.1
5	B/B	34	30	32	RUL-LUL	4.5	3.9	4.2
6	B/B	31	18	24.5	RUL-LUL	6.1	5.3	5.7
7	C/C	37	43	40	RUL-LUL	15.4	12.0	13.7
8	C/C	41.5	30	35.7	RLL-LLL	9.5	9.1	9.3
9	C/C	28	57	42.5	RLL-LUL	8.7	9.9	9.3
10	A/A	NLO	14.5	14.5	NLO-LUL	NLO	3.5	3.5
11	C/C	31.5	21	26.2	RUL-LUL	5.0	3.8	4.4
12	A/A	15.5	11	13.2	RUL-LUL	3.2	2.0	2.6
13	A/A	18.5	15	16.7	RUL-LUL	5.6	4	4.8
14	1–1 q-r/A	12	10.5	11.2	RUL-LUL	2.6	1.8	2.2
15	A/B	17	27	22	RUL-LUL	6.1	7.6	6.9
16	A/B	26	16.5	21.2	RUL-LUL	6.1	5.2	5.7
17	A/A	25.5	11	18.2	RUL-LUL	4.7	3.1	3.9

RUL: right upper lobe; LUL: left upper lobe; RLL: right lower lobe; LLL: left lower lobe; NLO: no large opacity.

All of the patients also demonstrated scattered micronodules throughout both lung fields, which were predominantly located in the upper lobes; moreover, calcifications in the pulmonary opacities were observed, and effects on the lymph nodes were noted to varying degrees.

### SUVmax and adenopathies

All of the patients demonstrated mediastinal lymphadenopathy with high metabolic activity. Additionally, 88.2% of patients exhibited hypermetabolic lymphadenopathy in other extrathoracic areas (such as in the supraclavicular, lower thoracic, and/or abdominal regions); a representative example is shown in Fig. 1c. The average SUVmax of the lymphadenopathies was 6.2 ± 1.5 (range: 4.1–10.8) (Table 2). A significant positive correlation was observed between the average SUVmax of the lymphadenopathies and the pulmonary opacities (*ρ *= 0.511, *P* = 0.036).

**Table 2:** Metabolic activity of thoracic and extrathoracic adenopathies measured by the SUV max.

Patient	Supraclavicular adenopathy	Right H-M adenophathy	Left H-M adenopathy	Subcarinal adenopathy	Lower thoracic adenopathy	Abdominal adenopathy	Average adenopathies
1	5.6	7.3	7.0	6.2	3.3		5.9
2		13.3	9.0	12.7		8.0	10.8
3	3.3	8.2	4.8	8.2	3.3	9.6	6.2
4	4.0	3.2	2.8	2.8		7.4	4.1
5	3.9	4.2	5.8	4.1	7.2	7.1	5.4
6	3.2	15.8	3.3	3.9	3.8	4.0	5.7
7	3.6	8.4	7.3	10.9		6.5	7.3
8		5.8	5.5	7.3			6.2
9		5.7		5.7		6.0	5.8
10	4.4	3.7	4.9	6.5			4.9
11	7.8	8.7	6.0	8.3	6.8	13.9	8.6
12	4.6	5.3	5.7	6.8		4.0	5.3
13	4.7	8.5	5.0	4.5		10.5	6.7
14		5.2	5.2	5.9			5.4
15	3.0	10.7	6.4	7.1	5.3	5.1	6.3
16	4.3	6.0	5.5	7.2	4.0	6.0	5.5
17		5.9	6.0	3.9	4.4		5.0

H-M: hilar-mediastinal. Blank spaces: no significant adenopathies.

### Relationships with occupational variables and other variables

We attempted to identify factors that may be associated with increased pulmonary uptake. We observed no correlation between the SUVmax and the years of exposure (*ρ *= 0.091, P = NS), the time elapsed since the cessation of exposure (*ρ *= 0.288, P = NS), the number of years from the start of exposure to the diagnosis of silicosis or PMF, or smoking history. However, we did identify a correlation between metabolic activity and opacity size (*ρ *= 0.747, *P* = 0.001), as well as a positive correlation between the ICOERD classification and the SUVmax (*ρ *= 0.697, *P* = 0.002). In patients classified as ICOERD category A, the SUVmax was 3.4 ± 1.0; moreover, the SUVmax was 6.5 ± 1.8 for patients classified as category B, and the SUVmax was 8.5 ± 3.4 for patients classified as category C. Similarly, a significantly positive correlation between the SUVmax and the ILO classification was observed (*ρ *= 0.626, *P* = 0.007).

With respect to the pulmonary function tests, the SUVmax was significantly and inversely correlated with various bronchial obstruction indices, such as the percentage of FEV_1_ (*ρ *= − 0.562, *P* = 0.019) and the FEV_1_/FVC ratio (*ρ *= − 0.565, *P* = 0.018); however, the SUVmax was not significantly correlated with the percentage of DLCOc (ρ = − .283, *P* = 0.348) or FVC (*ρ *= − 0.468, *P* = 0.058), although the latter correlation demonstrated borderline significance.

Linear regression analysis of all of the significant variables revealed that the best model included only the sizes of both pulmonary opacities as a covariate.

### SUVmax and biomarkers

**Fig. 2:**
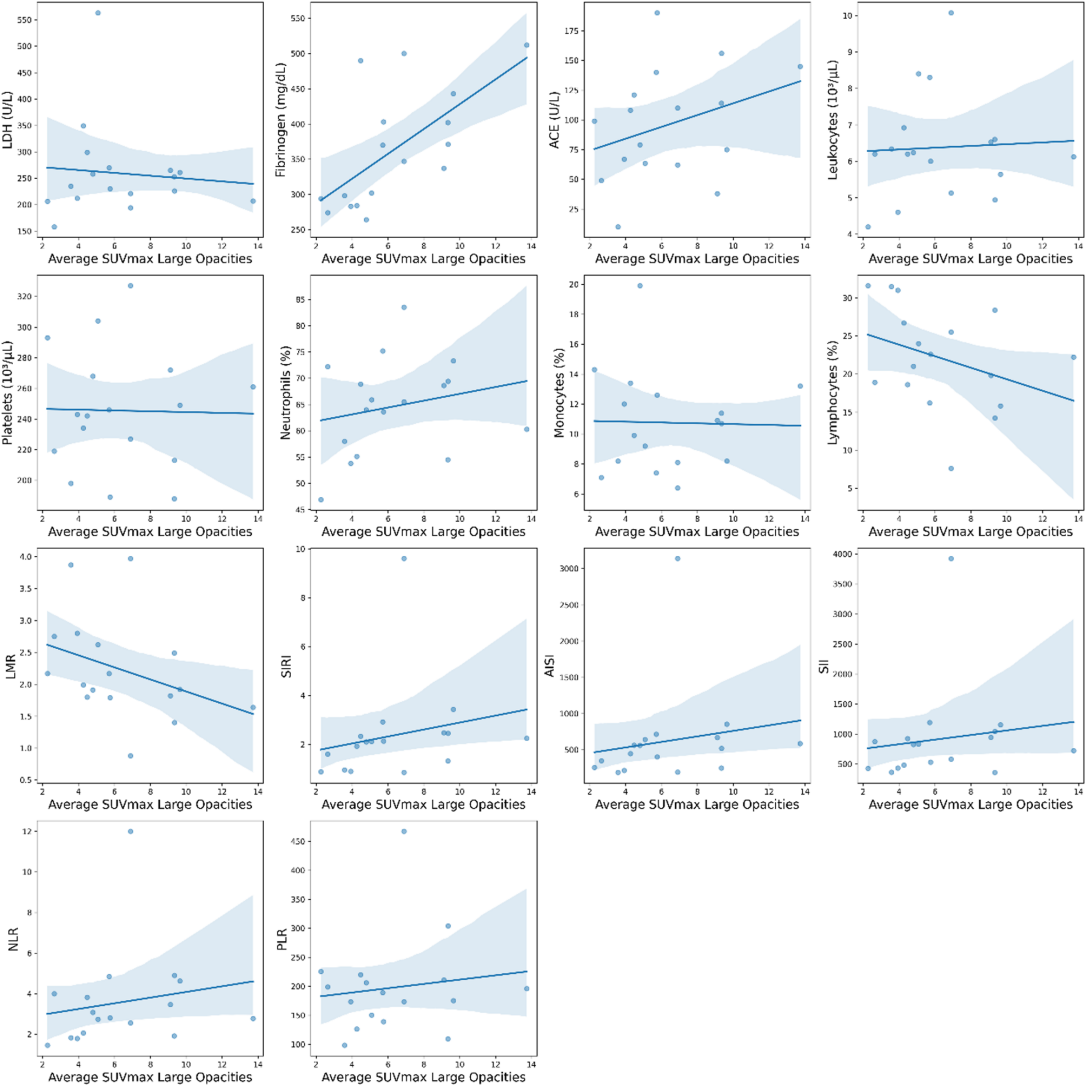
Regression plots for biomarkers against the average SUVmax of large opacities. Points represent the values obtained for each variable, while the line represents the best fit for the correlation between them (see Table 3 for detailed correlation values).

**Fig. 3:**
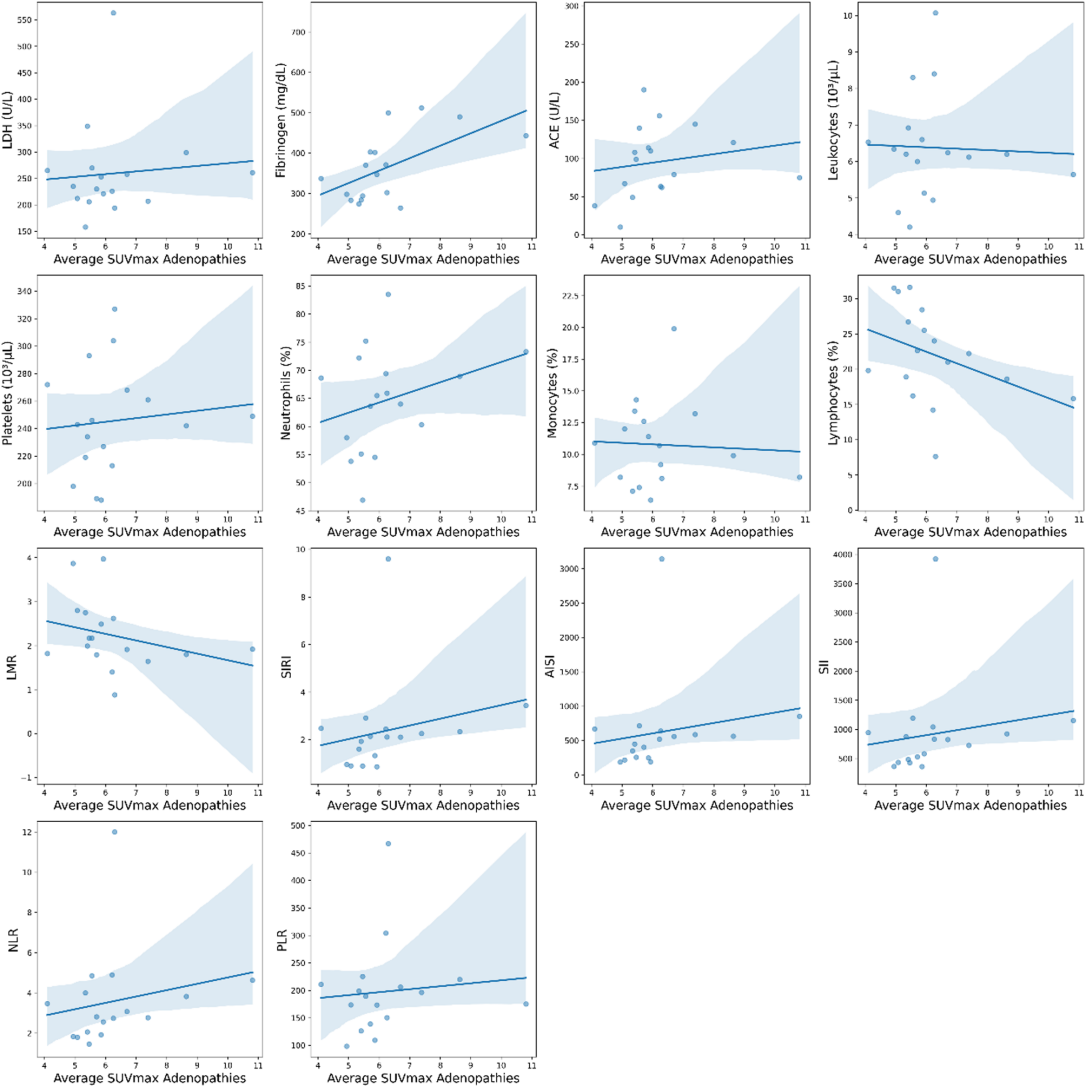
Regression plots for biomarkers against the average SUVmax of the lymphadenopathies. Points represent the values obtained for each variable, while the line represents the best fit for the correlation between them (see Table 3 for detailed correlation values).

We also explored the relationships between the SUVmax and several biomarkers and inflammatory indices (Table 3). The Spearman correlation analysis revealed that fibrinogen was, after correction for multiple comparisons, the only biomarker to show a statistically significant positive correlation with the SUVmax of the pulmonary conglomerates (*ρ *= 0.717, Benjamini–Hochberg adjusted *p* = 0.018). Regression plots for biomarkers against the average SUVmax for large opacities and adenopathies are shown in Figs. 2 and 3.

Moderate correlations were observed for blood leukocyte cells. The percentage of lymphocytes was negatively correlated with pulmonary metabolic activity (*ρ *= − 0.484) and lymphadenopathy metabolic activity (*ρ *= − 0.498).

**Table 3:** Relationships between the SUVmax and biomarkers and inflammatory indices.

Biomarkers and inflammatory index	Mean ± SD	Average SUVmax large opacities (ρ, *p*-values, Benjamini–Hochberg adjusted *p*-value )			Average SUVmax adenopathies (ρ, *p*-values, Benjamini–Hochberg adjusted *p*-value )		
LDH	259.24 ± 89.58	0,074	0,779	0,899	0,118	0,653	0,730
Fibrinogen	363.18 ± 83.19	0,717	0,001*	0,018*	0,593	0,012*	0,114
ACE	95.66 ± 46.66	0,389	0,123	0,205	0,404	0,107	0,170
Leucocytes (10^3^)	6,37 ± 1,46	0,048	0,855	0,916	− 0,012	0,963	0,981
Platelets (10^3^)	245,47 ± 39,36	0,013	0,959	0,959	0,255	0,323	0,384
Neutrophils (%)	64.62 ± 9.18	0,338	0,184	0,276	0,368	0,147	0,199
Monocytes (%)	10.75 ± 3.36	− 0,086	0,743	0,899	− 0,006	0,981	0,981
Lymphocytes (%)	22.09 ± 6.66	− 0,484	0,049*	0,143	− 0,498	0,042*	0,114
LMR	2.23 ± 0.79	-0,506	0,038*	0,143	− 0,482	0,050*	0,119
SIRI	2.36 ± 2	0,559	0,020*	0,098	0,436	0,080	0,169
AISI	621.21 ± 679.18	0,470	0,057	0,143	0,498	0,042*	0,114
SII	919.01 ± 820.98	0,390	0,122	0,205	0,407	0,105	0,170
NLR	3.57 ± 2.43	0,438	0,079	0,169	0,424	0,090	0,170
PLR	197.90 ± 85	0,085	0,747	0,899	0,284	0,269	0,340

LDH: lactate dehydrogenase; ACE: angiotensin converting enzyme.

In addition, moderate correlation was also observed between the SUVmax of pulmonary conglomerates and some systemic inflammatory indices, including SIRI (*ρ *= 0.559) and LMR (*ρ *= − 0.506). The SUVmax of the lymphadenopathies was again positively correlated with the AISI standard (*ρ *= 0.470), and NLR (*ρ *= 0.438). Although some of these reached nominal statistical significance (*p* < 0.05), none remained significant after adjustment for multiple testing, indicating that these associations should be interpreted with caution, given the limited sample size and type I error control.

### Metabolic activity and lymphocyte subsets

**Fig. 4:**
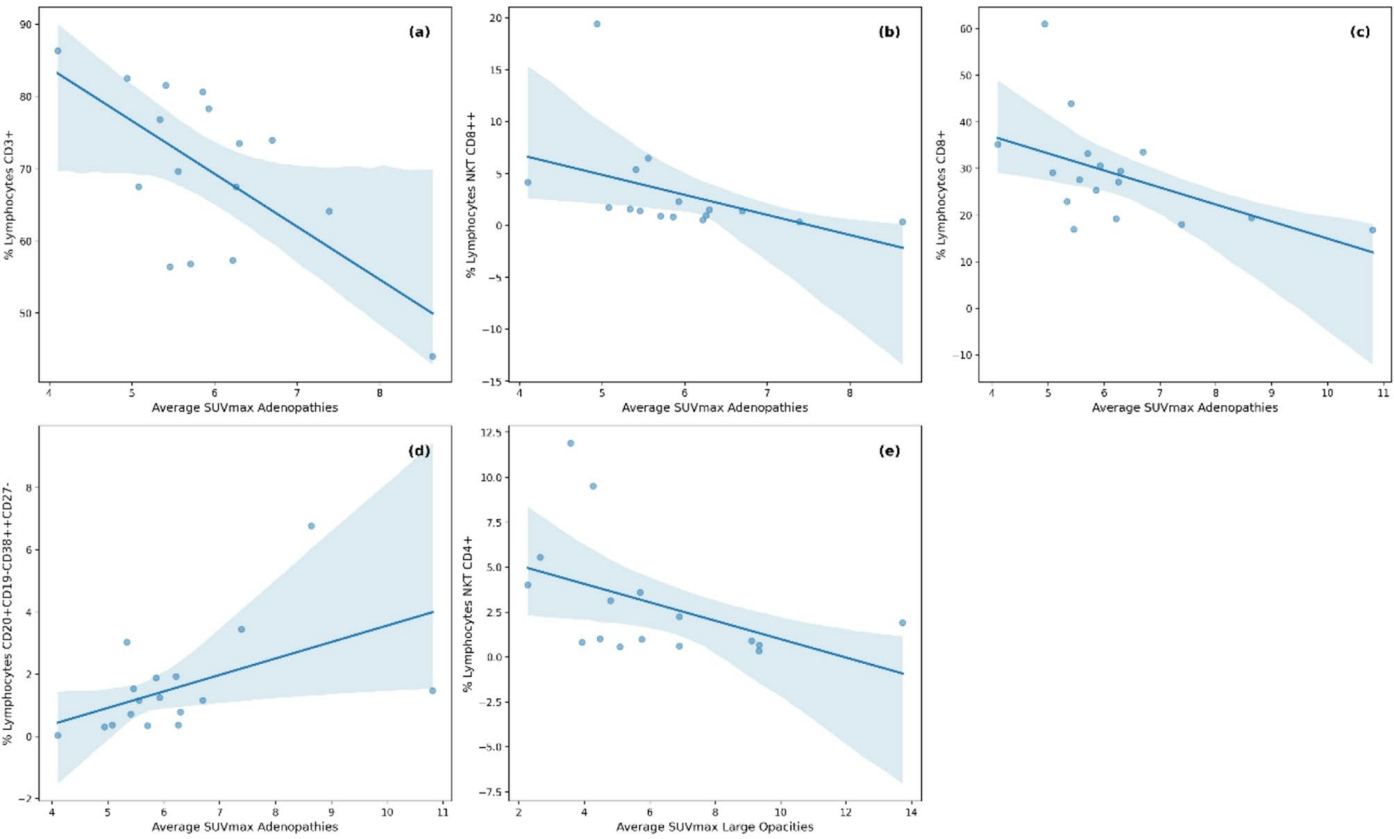
Regression plots for cell subsets against the average SUVmax of the lymphadenopathies and large opacities. (**a**) T-lymphocytes, (**b**) CD8^++^ NKT lymphocytes, (**c**) CD8^+^ T-lymphocytes, (**d**) CD20^+^CD19^−^CD38^++^CD27^−^ cells against the average SUVmax of adenophaties, and (**e**) Illustrates the regression plot for CD4^+^ NKT lymphocytes against the average SUVmax of the large opacities. Points represent the values obtained for each variable, while the line represents the best fit for the correlation between them (see Table 4 for detailed correlation values).

Upon further analysis of the specific lymphocyte subsets, the results revealed moderate to strong correlations with the SUVmax values (Table 4; Fig. 4). Notably, there was a marked negative correlation between total CD3^+^ cells, CD8^+^ cells, and CD8^+^NKT cells and lymphadenopathies (Fig. 4a−c), such that increased metabolic activity was associated with a decrease in these cell populations. Additionally, when the B-cell lineage was analyzed, plasma cells were significantly correlated with the SUVmax in the lymphadenopathies (Fig. 4d), although this correlation was not observed in the lung opacities. However, only CD4^+^NKT cells and the SUVmax in the pulmonary opacities were significantly correlated (Fig. 4e).

**Table 4:** Correlations between lymphocyte subsets and the SUVmax of large opacities and adenopathies.

Lymphocyte subsets (%) *	Average SUVmax large opacities		
	***ρ***	***p-value***	***Benjamini–Hochberg adjusted p-value***
CD4^+^NKT	− 0,611	0,012*	0,09
	**Average SUVmax Adenopathies**		
	***ρ***	***p-value***	***Benjamini–Hochberg adjusted p-value***
CD3^+^	− 0,522	0,038*	0,114
CD3^+^CD8^+^	− 0,720	0,002*	0,032*
CD8^+^NKT	− 0,517	0,034*	0,114
CD20^+^CD19^+^CD38^++^CD27^−^	0,542	0,025*	0,114

Only lymphocyte subsets with statistically significant correlations before correction are shown, and the remaining subsets are shown in supplementary Table S2.

## Discussion

Previous studies describing PET findings in silicosis have generally focused on isolated cases that were detected in the context of suspected lung cancer^11,12^. To our knowledge, this is the first prospective, systematic study that described the distribution and intensity of lung lesions and lymphadenopathy in patients with silicosis due to ES and strived to explore the relationships between biomarkers and both cellular populations and PET/CT metabolic activity, with the aim of better understanding the mechanisms of this disease.

One of the characteristics detected in our study involved intense metabolic activity, despite an average duration of more than 11 years since the cessation of exposure in the afflicted patients; this intense activity was observed in lung lesions, as well as in both mediastinal and extrathoracic lymphadenopathies. Thus, 70% of our patients exhibited hypermetabolic lymphadenopathy in the supraclavicular and abdominal regions. These results conflict with the findings demonstrated by six patients described by Reichert et al. who had classical silicosis, where only one patient demonstrated mediastinal lymphadenopathy, with none of the six patients exhibiting hypermetabolic extrathoracic lymphadenopathy^13^. The frequency of extrathoracic lymphadenopathy has been a surprising finding. Regarding supraclavicular lymphadenopathy, the supraclavicular nodes receive lymph drainage and cells from the lung^14^. Macrophages are recruited from the lymphatic vessels in inflamed areas and are transported to the lymph nodes^15^ In the context of abdominal lymphadenopathy, a retrograde flow mechanism through the thoracic duct has been described^16^. The increased aggressiveness of this type of silicosis^7^ and differences in the compositions and morphologies between natural and artificial compounds^17^ could explain these findings, although this issue remains unresolved. Moreover, the intense metabolic activity of lung lesions can be confused with neoplastic processes such as lung cancer^18^, and hypermetabolic mediastinal and extrathoracic lymphadenopathies may lead to an overestimation of cancer staging.

With respect to biomarkers, we selected those markers for which we have reported differences between patients with simple and complicated silicosis in a previous study^19^. Among the biomarkers, fibrinogen was observed to be highly significantly correlated with the SUVmax of the lung lesions. Fibrinogen is converted into fibrin, which induces cytokine expression and leukocyte recruitment^20^ and can be deposited in the lung tissue, thereby serving as a platform for inflammatory cells and fibroblasts in processes such as idiopathic pulmonary fibrosis^21,22^. Additionally, fibrin deposition is greater in the lungs of patients with idiopathic pulmonary fibrosis (IPF) than in those of healthy volunteers according to fibrin-specific PET^23^. Elevated fibrinogen levels were detected in our previous study involving patients with simple silicosis, and even higher levels were detected in patients with complicated silicosis^19^. Our current research revealed a strong correlation between fibrinogen and metabolic activity in complicated silicosis, thus further supporting a possible role of the coagulation system in the development of progressive massive fibrosis.

We observed no correlation between the SUVmax and either exposure time or the time since the cessation of exposure; however, we did identify a strong positive correlation between the size of the lung lesion and its metabolic activity.

The progression rate is related to the silicon (Si) content in the nodules; specifically, a higher Si content corresponds to a faster progression, even after exposure has ended^24^. Alveolar macrophages phagocytose silica particles, thereby causing lysosomal damage and activation of the NLRP3 inflammasome, which leads to the release of multiple inflammatory and proinflammatory cytokines. These cytokines, along with ROS and RNS intermediaries, drive the cycle of apoptotic cell death and fibrosis^25^. Silica is released into the extracellular space; in conjunction with cell death components, this compound results in a progressive increase in inflammatory and fibrogenic cells, thereby leading to increased metabolic activity and the resulting size of the silicotic conglomerate. The elucidation of the involved cells could be highly important in identifying therapeutic targets to help in disrupting this vicious cycle.

The relationships between specific blood cells and various outcomes of interstitial diseases are becoming increasingly evident and are a subject of investigation, as they may serve as accessible and easy-to-obtain biomarkers with prognostic value; additionally, they can even provide clues for potential therapeutic targets. For example, Kreuter^26^ reported a negative correlation between the progression of IPF, hospitalizations, mortality and the number of lymphocytes. Furthermore, Achaiah et al. reported that neutrophils in the blood and lymphopenia can function as prognostic indicators of IPF progression^27^.

In our study, the percentage of lymphocytes was negatively correlated with both pulmonary metabolic activity (*ρ * = − 0.484) and lymphadenopathy (*ρ * = − 0.498), thereby indicating that an increased SUVmax was associated with fewer lymphocytes. Moderate correlations were also observed with certain inflammatory indices (such as LMR, SIRI, and AISI), which involve other blood cells (such as monocytes, neutrophils, and platelets) in ratio calculations with the lymphocyte count. A decrease in lymphocytes and changes in these inflammatory indices have been detected in patients with complicated silicosis compared with those with simple silicosis and healthy controls^19^. Decreased lymphocytes and altered LMR values have also been detected among silica-exposed individuals without disease and in patients with silicosis^28^.

The relationship between decreased lymphocytes and the intensity of metabolic activity in both pulmonary opacities and lymphadenopathy is an interesting finding that has not been previously described. In the different lymphocyte subgroups, a decrease in CD4^+^ natural killer T (NKT) cells was significantly correlated with increased metabolic activity in the pulmonary opacities, whereas a decrease in CD3^+^ and CD8^+^ NKT cells was correlated with increased metabolic activity in lymphadenopathy. In an experimental model of silicosis, Davis et al. observed the accumulation of lymphocytes in nodules and lymphadenopathy. The detected lymphocytes were predominantly CD4^+^ T cells; however, numerous CD8^+^ T cells, NKT cells, and CD4-γδ-TCR^+^ T cells were also present^29^. NKT cells are considered to function as a bridge between innate and adaptive immunity, whereby they function in both protective and pathogenic roles; moreover, they have been reported to contribute to various diseases, such as autoimmune diseases, infections, and cancer^30,31^. CD4^+^ NKT cell numbers are reduced in patients with multiple sclerosis, and they can improve the state of the disease by directing immune responses toward a Th2 response^32^. The expansion of CD8^+^ NKT cells has been reported in chronic immune activation, such as sarcoidosis^33^; additionally, CD8^+^ NKT cells are considered to be the most efficient transactivators of CD8^+^ T cells^34^. This could explain why we observed a negative correlation between CD8^+^ T cells or CD8^+^ NKT cells and the SUVmax of lymphadenopathy, with a strong correlation of the latter.

Finally, we observed that memory B cells and plasmablasts or plasma cells were positively correlated with the SUVmax of lymphadenopathy. Complex relationships between B cells and NKT cells have been previously described, which could explain the observed relationships between the percentages of these subpopulations and the SUVmax values of large opacities and lymphadenopathy^35,36^. The hypothesis regarding this relationship is that silica-activated macrophages produce interleukins that attract and activate lymphocytes, which correspondingly attract and activate more macrophages^29^. In this manner, some lymphocyte subsets could play different roles in inflammatory activity, thus causing an imbalance in the global response and favoring the progression of the disease.

One limitation of this study is the lack of knowledge regarding the inhaled silica load; moreover, the correlation of this load with the exposure time may be inaccurate, as some of our investigated workers experienced intense exposure over short periods of time^37^. Despite being, to our knowledge, the largest prospective series on silicosis and the only one specifically addressing exposure to ES, a limitation of this study is the small sample size (*n* = 17). This constraint limits the generalizability of the findings, particularly the observed correlations between biomarkers, immune cell populations, and metabolic activity. Therefore, future research with larger cohorts and longitudinal follow-up is essential to confirm and expand upon these results. A further potential limitation is whether other PET parameters, such as metabolic tumor volume (MTV) and total lesion glycolysis (TLG), may be more useful for measuring systemic inflammatory activity. However, the application of these volumetric parameters in silicosis may be imprecise due to the difficult delimitation of the metabolic volume, which is caused by the multiplicity of diffuse micronodules (many of which are smaller or borderline centimeter-sized), and the heterogeneity of uptake. Therefore, we used the SUVmax as a parameter because of its repeatability and correlation with systemic biomarkers^38^. In addition, our study revealed a strong correlation with systemic inflammatory markers such as fibrinogen. Despite these limitations, we have demonstrated very interesting findings that may help to improve our understanding of the disease mechanisms and the anatomical distribution of hypermetabolic lymphadenopathy. Recently, it has been suggested that lung function assessed via the FVC is not a sensitive measure of progression in most types of pneumoconiosis^39^; thus, PET/CT could be an alternative method for evaluating the efficacy of drugs in this disease.

## Conclusions

In summary, ES silicosis produces intense metabolic activity both in the lungs and in thoracic and extrathoracic lymphadenopathies, and this activity persists even years after exposure has ceased. When considering that silica is a Group 1 carcinogen and responsible for the potential coexistence of lung cancer, an understanding of the topographic distribution of hypermetabolic lymphadenopathy related to silica exposure is important to avoid the overstaging of neoplastic processes via PET. Additionally, the strong relationships that were demonstrated between metabolic activity and fibrinogen provide an avenue for exploring new drugs that have not yet been tested in this disease, such as monoclonal antibodies antagonizing factor XIIa, which are currently being investigated in idiopathic pulmonary fibrosis (CSL312 Safety, Pharmacokinetics, and Pharmacodynamics in Idiopathic Pulmonary Fibrosis; ClinicalTrials.gov ID NCT05130970). Lastly, the preliminary links found between metabolic activity and lymphocyte subsets may warrant additional studies on artificial stone silicosis, with this work serving as a potential starting point.

## Methods

### Study population

Seventeen patients who were diagnosed with silicosis and PMF caused by ES were included in this study. These patients are part of a cohort being monitored at Puerta del Mar University Hospital (Cádiz, Spain). All of the patients were diagnosed with silicosis based on their history of exposure to ES and radiological findings, chest radiography and high-resolution computed tomography (HRCT); moreover, some patients were further confirmed via lung or lymph node biopsies. This study was approved by the Research Ethics Committee of the province of Cádiz and the Spanish Agency of Medicines and Medical Devices (Eudra CT 2021-002701-94; date: 08/13/2021). All of the research was performed following the Declaration of Helsinki in accordance with relevant guidelines/regulations, and informed consent was obtained from all of the participants.

A control group was excluded from this study for two main reasons. First, due to ethical reasons, we avoided subjecting healthy individuals to unnecessary testing. Second, we did not expect to obtain metabolically active CT/PET images from healthy individuals with healthy lungs. However, blood markers have been extensively investigated in this cohort compared with a healthy group and a group of patients with simple silicosis^19,40^.

The following inclusion criteria were used: male patients aged 18–65 years with at least five years of exposure to ES and diagnosed with silicosis and PMF. The following exclusion criteria were used: active smokers; other diseases affecting silicosis progression (including cancer, HIV, hepatitis, liver failure or renal failure); active infectious disease; and immunosuppressive, immunomodulatory, antifibrotic or biological therapies. Only patients taking prednisone at doses of 20 mg/day or lower were included in the study. The first patient was enrolled in November 2021, and the last patient was enrolled in September 2022.

Eleven patients were diagnosed with ES silicosis between 2010 and 2011 and subsequently ceased exposure to ES. The remaining patients were diagnosed at later times, although their exposures had ceased years earlier. At the time of diagnosis, 11 patients had simple silicosis, whereas 6 patients were diagnosed with complicated silicosis PMF category A.

The sociodemographic characteristics of the patients at the time of their inclusion in the study are described in Table 5. All of the patients had worked for small companies involved in cutting, polishing and installing kitchens and bathroom countertops.

**Table 5:** Sociodemographic and labor data of the participants and pulmonary function values.

	Mean ± SD
Age (years)	44 ± 5.4
Starting Exposure Age	21.6 ± 5.1
Duration of Exposure (years)	10.9 ± 3.2
Years from start of exposure to diagnosis of silicosis.	13.0 ± 4.2
Years from start of exposure to diagnosis of PMF.	16.9 ± 3.6
Years from cessation of exposure to blood extraction and PET/CT (study)	11.6 ± 1.6
Never-smoker*	9 (53%)
Ex-smoker*	8 (47%)
Pack-years	7.1 ± 4.8
FVC (mL)	3914 ± 968
FVC (%)	79.1 ± 17.3
FEV_1_ (mL)	2796 ± 899
FEV_1_ (%)	70.8 ± 21.7
FEV_1_/FVC	0.69 ± 0.10
DLCOc (mmol/min/kPa)	8.6 ± 1.5
DLCOc (%)	85.3 ± 14.8

Note: FEV_1_: Forced expiratory volume in the first second, forced vital capacity (FVC); DLCOc: Diffuse capacity of the lung for carbon monoxide. *Number of cases (percentage).

A comprehensive clinical interview, respiratory function tests, blood sampling, and a PET/CT scan were performed on all of the patients, with all of these procedures being consecutively performed on the same morning from 8:30 a.m. to 12:30 p.m. Respiratory function tests were performed by trained personnel using an EasyOne Pro system (ndd Medizintechnik AG, Zurich, Switzerland) following international guidelines^41,42^. The radiological classification of silicosis was based on the International Labor Organization (ILO) classification for chest radiographs^43^. The CT procedure, which was integrated into the PET/CT scans, was performed using 2 mm slices in the thoracic region, and the classification of the images was performed via the ICOERD classification^44^. Large opacities were defined when the mean diameter (measured in two perpendicular axes) exceeded 1 cm. Three experts independently interpreted the chest X-rays and HRCT scans, and they used the ICOERD classification to categorize the large opacities as categories A, B, or C.

### Acquisition of PET/CT images and image analysis

PET/CT scans were performed via a Biograph mCT 20 Excel hybrid scanner (Siemens^®^) with time-of-flight (TOF) technology, following a minimum 6-hour fasting period and relative rest on the day before the scan. Fifty-five to sixty-five minutes after the administration of ^18^F-FDG, a low-dose CT scan (Somatom Definition AS 20) was acquired with CARE Dose 4D software (80 KW, 20 slices per rotation, 120 kVp, 35 mA and 2 mm thickness, without oral or intravenous contrast administration and without breath-holding). PET scans were performed from the vertex to the proximal third of the lower extremities, with the arms being positioned overhead. Images were obtained with and without attenuation correction, and iterative reconstruction was performed via 6 iterations, 21 segments, and a Gaussian filter of 500 mm in the axial, coronal, and sagittal planes.

The ^18^F-FDG dose was calculated based on patient weight (MBq/kg), with a standard dose of 3.9 MBq/kg being used [259–381]. The acquisition time varied between 2 and 3.5 min per bed position, and blood glucose levels did not exceed 200 mg/dL. PET/CT scans were performed under consistent conditions of dose, injection-to-acquisition time, and reconstruction parameters.

The PET/CT findings were classified as pulmonary, hilar-mediastinal (H-M) or extrathoracic. Lesions were considered to be pathological when ^18^F-FDG uptake exceeded that of the thoracic descending aorta. All of the hypermetabolic large opacities in the pulmonary parenchyma were evaluated, and the opacity with the highest uptake was selected from each lung.

For thoracic or extrathoracic lymph nodes, the largest and most metabolically active nodes were highlighted. All of the examination results were independently reviewed by two experienced nuclear medicine physicians, and a third expert opinion was requested in any cases of uncertainty.

For quantitative PET/CT analysis, the volume of interest (VOI) was manually defined, and the maximum standard uptake value (SUVmax) was obtained from all of the regions^45^. We selected the VOI with the highest activity from each lung, with an uptake threshold of 40%. The average SUVmax of both lesions was subsequently calculated.

### Analysis of systemic Inflammatory indices and lymphocyte populations

Blood samples were collected after fasting and immediately processed for biochemical and hematological analyses. Standard hematological parameters were automatically analyzed via an XN-1000 analyzer (Sysmex, Germany). The following inflammatory indices were calculated: neutrophil-to-lymphocyte ratio (NLR), platelet-to-lymphocyte ratio (PLR), lymphocyte-to-monocyte ratio (LMR), neutrophil × platelet-to-lymphocyte ratio (known as the systemic immune-inflammation index, or SII), neutrophil × monocyte-to-lymphocyte ratio (known as the systemic inflammation response index, or SIRI), and neutrophil × monocyte × platelet-to-lymphocyte ratio (known as the aggregate index of systemic inflammation, or AISI).

A detailed immune lymphocyte profile was performed on 150 µL of fresh peripheral blood via cell surface immunostaining with the corresponding fluorochrome-conjugated antibodies for 15 min in the dark (Supplementary Table S1). After the blood was treated with 2 mL of lysis solution (Becton Dickinson; San Jose, CA, USA) for 5 min in the dark, the samples were analyzed via flow cytometry, and the results are reported as percentages of total lymphocytes. For intracellular staining, after surface staining, the cells were fixed and permeabilized with the Cytofix/Cytoperm Kit (Becton Dickinson). Afterwards, intracellular fluorochrome-conjugated antibodies (anti-GATA3, anti-RORγT and anti-t-BET) were added, and the samples were incubated for 30 min in the dark, washed, centrifuged, and analyzed via flow cytometry. The analyzed lymphocyte subsets are shown in Supplementary Table S2.

### Statistical analysis

The results are expressed as the means and standard deviations (SDs) or as the number of cases and percentages. Given that not all variables followed a normal distribution as assessed by the Kolmogorov–Smirnov test (*p* < 0.05 for several variables), a correlation analysis was performed to evaluate the relationship between biomarkers and lymphocyte subpopulations with metabolic activity in the lungs and lymphadenopathy regions, as measured by average SUVmax values. Spearman’s rank correlation coefficient (ρ) was used due to the small sample size and its robustness to outliers. Variables with missing values were excluded pairwise from each respective correlation calculation. Statistical significance was set at *p* < 0.05. To account for multiple comparisons, p-values were adjusted using the Benjamini–Hochberg procedure to control the false discovery rate (FDR) and reduce the likelihood of Type I errors^46^. All analyses were performed using Python and SPSS software.

## Electronic supplementary material

Below is the link to the electronic supplementary material.

Supplementary Material 1

## Data Availability

The data are not publicly available due to privacy or ethical restrictions. The data that support the findings of this study are available upon request from the corresponding author for researchers who meet the criteria for confidential data access, as stipulated by participant informed consent and the Institutional Research Ethics Committee of the province of Cadiz, Spain. Data requests can be made to this Ethics committee via this email: ceic.hpm.sspa@juntadeandalucia.es.

## References

[1] Hoy, R. F. et al. Current global perspectives on silicosis-Convergence of old and newly emergent hazards. *Respirology* **27**, 387–398. 10.1111/resp.14242 (2022). 35302259 10.1111/resp.14242 PMC9310854

[2] Fazio, J. C. et al. Silicosis among immigrant engineered stone (Quartz) countertop fabrication workers in California. *JAMA Intern. Med.* **183**, 991–998. 10.1001/jamainternmed.2023.3295 (2023). 37486642 10.1001/jamainternmed.2023.3295 PMC10366949

[3] Martinez Gonzalez, C. et al. Silicosis in artificial quartz conglomerate workers. *Arch. Bronconeumol. (Engl Ed)*. **55**, 459–464. 10.1016/j.arbres.2019.01.017 (2019). 30879876 10.1016/j.arbres.2019.01.017

[4] Kromhout, H., van Tongeren, M. & Cherrie, J. W. Should engineered stone products be banned? *Occup. Environ. Med.* **81**, 329–330. 10.1136/oemed-2024-109708 (2024). 39107095 10.1136/oemed-2024-109708

[5] Leon-Jimenez, A. New etiological agents of silicosis. *Arch. Bronconeumol.* **59**, 479–480. 10.1016/j.arbres.2023.03.003 (2023). 36967343 10.1016/j.arbres.2023.03.003

[6] Ramkissoon, C. et al. Understanding the pathogenesis of engineered stone-associated silicosis: the effect of particle chemistry on the lung cell response. *Respirology* **29**, 217–227. 10.1111/resp.14625 (2024). 38043119 10.1111/resp.14625

[7] Wu, N., Xue, C., Yu, S. & Ye, Q. Artificial stone-associated silicosis in china: A prospective comparison with natural stone-associated silicosis. *Respirology* **25**, 518–524. 10.1111/resp.13744 (2020). 31828940 10.1111/resp.13744 PMC7187561

[8] Leon-Jimenez, A. et al. Artificial stone silicosis: rapid progression following exposure cessation. *Chest* **158**, 1060–1068. 10.1016/j.chest.2020.03.026 (2020). 32563682 10.1016/j.chest.2020.03.026

[9] Li, R., Kang, H. & Chen, S. From basic research to clinical practice: considerations for treatment drugs for silicosis. *Int. J. Mol. Sci.* **24**10.3390/ijms24098333 (2023). 10.3390/ijms24098333 PMC10179659 37176040

[10] Capitanio, S., Nordin, A. J., Noraini, A. R. & Rossetti, C. PET/CT in nononcological lung diseases: current applications and future perspectives. *Eur. Respir Rev.* **25**, 247–258. 10.1183/16000617.0051-2016 (2016). 27581824 10.1183/16000617.0051-2016 PMC9487216

[11] del Castillo-Otero, D., Hidalgo-Molina, R. R. J. & Conde- Sánchez, A. Pajares -Vinardell M and León- Jiménez A. Positron emission tomography in artificial stone silicosis. *J. Lung Pulmonary Respiratory Res.* **11**, 37–39. 10.15406/jlprr.2024.11.00316 (2024).

[12] Matar, E. et al. Complicated silicosis resulting from occupational exposure to engineered stone products. *Med. J. Aust*. **206**, 385–386. 10.5694/mja16.00257 (2017). 28490299 10.5694/mja16.00257

[13] Reichert, M. & Bensadoun, E. S. PET imaging in patients with coal workers pneumoconiosis and suspected malignancy. *J. Thorac. Oncol.* **4**, 649–651. 10.1097/JTO.0b013e31819d4778 (2009). 19395909 10.1097/JTO.0b013e31819d4778

[14] Banjar, F. K. & Wilson, A. M. *In Anatomy, Head and Neck, Supraclavicular Lymph Node* (StatPearls Publishing, 2025). 31335020

[15] Zhou, C., Sun, T., Dong, Z., Lu, F. & Li, B. The interplay between lymphatic vessels and macrophages in inflammation response. *FASEB J.* **38**, e23879. 10.1096/fj.202400160RR (2024). 39162663 10.1096/fj.202400160RR

[16] Ryu, W. et al. Abdominal lymph node metastasis by lymphatic spread through the thoracic duct in patients with non-small-cell lung cancer. *Thorac. Cancer*. **12**, 2078–2084. 10.1111/1759-7714.14014 (2021). 34033231 10.1111/1759-7714.14014 PMC8287020

[17] Ramkissoon, C. et al. Characterisation of dust emissions from machined engineered stones to understand the hazard for accelerated silicosis. *Sci. Rep.* **12**, 4351. 10.1038/s41598-022-08378-8 (2022). 35288630 10.1038/s41598-022-08378-8 PMC8921240

[18] Kang, S. R. & Rho, J. Y. Progressive massive fibrosis mimicking lung cancer: two case reports with potentially useful CT features for differential diagnosis. *J. Korean Soc. Radiol.* **83**, 1175–1181. 10.3348/jksr.2021.0185 (2022). 36276214 10.3348/jksr.2021.0185 PMC9574288

[19] Garcia-Nunez, A. et al. Inflammatory indices obtained from routine blood tests show an inflammatory state associated with disease progression in engineered stone silicosis patients. *Sci. Rep.* **12**, 8211. 10.1038/s41598-022-11926-x (2022). 35581230 10.1038/s41598-022-11926-x PMC9114118

[20] Jennewein, C. et al. Novel aspects of fibrin(ogen) fragments during inflammation. *Mol. Med.* **17**, 568–573. 10.2119/molmed.2010.00146 (2011). 21210072 10.2119/molmed.2010.00146 PMC3105136

[21] Bargagli, E. et al. Serum analysis of coagulation factors in IPF and NSIP. *Inflammation* **37**, 10–16. 10.1007/s10753-013-9706-z (2014). 23912648 10.1007/s10753-013-9706-z

[22] Schuliga, M., Grainge, C., Westall, G. & Knight, D. The fibrogenic actions of the coagulant and plasminogen activation systems in pulmonary fibrosis. *Int. J. Biochem. Cell. Biol.* **97**, 108–117. 10.1016/j.biocel.2018.02.016 (2018). 29474926 10.1016/j.biocel.2018.02.016

[23] Munchel, J. K. et al. Fibrin-Positron emission tomography imaging reveals ongoing lung injury in idiopathic pulmonary fibrosis. *Am. J. Respir Crit. Care Med.* **210**, 514–517. 10.1164/rccm.202312-2357LE (2024). 38843499 10.1164/rccm.202312-2357LE PMC11351804

[24] Leon-Jimenez, A. et al. Compositional and structural analysis of engineered stones and inorganic particles in silicotic nodules of exposed workers. *Part. Fibre Toxicol.* **18**10.1186/s12989-021-00434-x (2021). 10.1186/s12989-021-00434-x PMC8607701 34809667

[25] Vanka, K. S. et al. Understanding the pathogenesis of occupational coal and silica dust-associated lung disease. *Eur. Respir Rev.* **31**10.1183/16000617.0250-2021 (2022). 10.1183/16000617.0250-2021 PMC9724915 35831008

[26] Kreuter, M. et al. Monocyte count as a prognostic biomarker in patients with idiopathic pulmonary fibrosis. *Am. J. Respir Crit. Care Med.* **204**, 74–81. 10.1164/rccm.202003-0669OC (2021). 33434107 10.1164/rccm.202003-0669OC PMC8437112

[27] Achaiah, A. et al. Increased monocyte level is a risk factor for radiological progression in patients with early fibrotic interstitial lung abnormality. *ERJ Open. Res.* **8**10.1183/23120541.00226-2022 (2022). 10.1183/23120541.00226-2022 PMC9251369 35795307

[28] Lombardi, E. M. S., Mizutani, R. F., Terra-Filho, M., Ubiratan & de Paula Biomarkers related to silicosis and pulmonary function in individuals exposed to silica. *Am. J. Ind. Med.* **66**, 984–995. 10.1002/ajim.23528 (2023). 37615855 10.1002/ajim.23528

[29] Davis, G. S., Holmes, C. E., Pfeiffer, L. M. & Hemenway, D. R. Lymphocytes, lymphokines, and silicosis. *J. Environ. Pathol. Toxicol. Oncol.* **20** (Suppl 1), 53–65 (2001). 11570674

[30] Nelson, A., Lukacs, J. D. & Johnston, B. The current landscape of NKT cell immunotherapy and the hills ahead. *Cancers (Basel)*. **13**10.3390/cancers13205174 (2021). 10.3390/cancers13205174 PMC8533824 34680322

[31] Dhodapkar, M. V. & Kumar, V. Type II NKT cells and their emerging role in health and disease. *J. Immunol.* **198**, 1015–1021. 10.4049/jimmunol.1601399 (2017). 28115591 10.4049/jimmunol.1601399 PMC5300729

[32] Ahmadi, A. et al. The role of NK and NKT cells in the pathogenesis and improvement of multiple sclerosis following disease-modifying therapies. *Health Sci. Rep.* **5**, e489. 10.1002/hsr2.489 (2022). 35229046 10.1002/hsr2.489 PMC8865072

[33] Naccache, J. M. et al. Increasing level of CD56 + T-cells in peripheral blood in sarcoidosis. *Eur. Respir J.* **27**, 654. 10.1183/09031936.06.00129505 (2006). 16507870 10.1183/09031936.06.00129505

[34] Lin, H., Nieda, M., Rozenkov, V. & Nicol, A. J. Analysis of the effect of different NKT cell subpopulations on the activation of CD4 and CD8 T cells, NK cells, and B cells. *Exp. Hematol.* **34**, 289–295. 10.1016/j.exphem.2005.12.008 (2006). 16543063 10.1016/j.exphem.2005.12.008

[35] Doherty, D. G., Melo, A. M., Moreno-Olivera, A. & Solomos, A. C. Activation and regulation of B cell responses by invariant natural killer T cells. *Front. Immunol.* **9**, 1360. 10.3389/fimmu.2018.01360 (2018). 29967611 10.3389/fimmu.2018.01360 PMC6015876

[36] Leadbetter, E. A. & Karlsson, M. C. I. Invariant natural killer T cells balance B cell immunity. *Immunol. Rev.* **299**, 93–107. 10.1111/imr.12938 (2021). 33438287 10.1111/imr.12938 PMC8485762

[37] Perez-Alonso, A., Gonzalez-Dominguez, M. E., Novalbos-Ruiz, J. P., Leon-Jimenez, A. & Cordoba-Dona, J. A. Artificial stone silicosis: accumulation of errors in the resurgence of an occupational disease: A qualitative study. *Work* **70**, 433–442. 10.3233/WOR-213582 (2021). 34633345 10.3233/WOR-213582

[38] Donnelly, R. et al. Meta-analysis of [(18)F]FDG-PET/CT in pulmonary sarcoidosis. *Eur. Radiol.* **35**, 2222–2232. 10.1007/s00330-024-10949-4 (2025). 39044038 10.1007/s00330-024-10949-4 PMC11913913

[39] Thiruvarudchelvan, A., Hart-Brown, L., Bloch, M. & Yates, D. The nintedanib in progressive pneumoconiosis study (NiPPs): early data from Australia. *Eur. Respir. J.* **62**, PA3800. 10.1183/13993003.congress-2023.PA3800 (2023).

[40] Jimenez-Gomez, G. et al. Analysis of immune cell subsets in peripheral blood from patients with engineered stone Silica-Induced lung inflammation. *Int. J. Mol. Sci.* **25**10.3390/ijms25115722 (2024). 10.3390/ijms25115722 PMC11171478 38891910

[41] Graham, B. L. et al. Standardization of spirometry 2019 update. An official American thoracic society and European respiratory society technical statement. *Am. J. Respir Crit. Care Med.* **200**, e70–e88. 10.1164/rccm.201908-1590ST (2019). 31613151 10.1164/rccm.201908-1590ST PMC6794117

[42] Graham, B. L. et al. 2017 ERS/ATS standards for single-breath carbon monoxide uptake in the lung. *Eur. Respir J.* **49**10.1183/13993003.00016-2016 (2017). 10.1183/13993003.00016-2016 28049168

[43] International Labour Office. *Geneva, S. I. L. O* (International Labour Office, 2011).

[44] Suganuma, N. et al. Reliability of the proposed international classification of high-resolution computed tomography for occupational and environmental respiratory diseases. *J. Occup. Health*. **51**, 210–222. 10.1539/joh.l8030 (2009). 19372629 10.1539/joh.l8030

[45] Boellaard, R. et al. FDG PET/CT: EANM procedure guidelines for tumour imaging: version 2.0. *Eur. J. Nucl. Med. Mol. Imaging*. **42**, 328–354. 10.1007/s00259-014-2961-x (2015). 25452219 10.1007/s00259-014-2961-x PMC4315529

[46] Benjamini, Y. & Hochberg, Y. Controlling the false discovery rate: A practical and powerful approach to multiple testing. *J. Roy. Stat. Soc.: Ser. B (Methodol.)*. **57**, 289–300. 10.1111/j.2517-6161.1995.tb02031.x (1995).

