# Supplementary material for: High metabolic activity in positron emission tomography and systemic inflammation occurring years after exposure cessation in engineered stone silicosis
Source: Sci Rep. 2025 Jul 14;15:25364. doi: 10.1038/s41598-025-10562-5 (PMC12260104; doi:10.1038/s41598-025-10562-5)
Supplement: Supplementary file 1 — Supplementary Material 1 [file 41598_2025_10562_MOESM1_ESM.pdf]

Supplemental material

Table S1. Antibodies used for flow cytometry analysis

antiCD45 V500 (ref. 560777, Becton Dickinson)
antiCD3 APC-H7 (ref. 641415, Becton Dickinson)
antiCD4 V450 (ref. 651849, Becton Dickinson)
antiCD8 PerCP (ref. 21810085X2, ImmunoTools)
antiCD56 APC (ref. 555518, Becton Dickinson)
antiCD19 PerCP CY7 (ref. 341113, Becton Dickinson)
antiCD27 PE (ref. 340425, Becton Dickinson)
antiCD16 FITC (ref. 335035, Becton Dickinson)
antiCD45RA FITC (ref. 335039, Becton Dickinson)
antiCD45RO PE (ref. 347967, Becton Dickinson)
antiCD127 PerCP CY5.5 (ref. 351322, Biolegend)
antiCD25 PerCP CY7 (ref. 335824, Becton Dickinson)
antiGATA3* FITC (ref. 130-120-061, Miltenyi)
antiRORγt* APC (ref. 130-123-840, Miltenyi)
antit-BET* PE (ref. 130-121-340, Miltenyi)
antiCD38 FITC (ref. 21270383X2 ImmunoTools)
antiCD19 APC (ref. 21270196X2 ImmunoTools)

*intracytoplasmic antibodies

Table S2. Lymphocytes subsets analyzed.

Cell markers tested		Immune cell phenotype
**B cells**		
	$CD19^{+}$	Total B cells
	$CD19^{+}$ $CD27^{+}$	Memory B cells
	$CD38^{++}$ $CD19^{+/-}$	Plasma cells
**NK cells**		
	$CD3^{-}$ $CD56^{+}$	Total NK cells
	$CD3^{-}$ $CD56^{+}$ $CD16^{+}$	Cytotoxic NK cells
	$CD3^{-}$ $CD56^{++}$ $CD16^{-}$	Naïve/Regulatory NK cells
**T cells**		
	$CD3^{+}$	Total T cells
	$CD3^{+}$ $CD4^{+}$	Total helper T cells
	$CD3^{+}$ $CD8^{+}$	Total cytolytic T cells
	$CD3^{+}$ $CD4^{+}$ $CD8^{+}$	Pro-T cells
	$CD3^{+}$ $CD4^{-}$ $CD8^{-}$	Naïve T cells
**Th cell subsets**		
	$CD3^{+}$ $CD4^{+}$ $CD45RA^{+}$ $CD45RO^{-}$	Naïve helper T cells
	$CD3^{+}$ $CD4^{+}$ $CD45RA^{+}$ $CD45RO^{+}$	Memory helper T cells
	$CD3^{+}$ $CD4^{+}$ $CD25^{++}$ $CD127^{-}$	Regulatory T cells ($CD4^{+}$)
	$CD3^{+}$ $CD4^{-}$ $CD25^{+}$ $CD127^{+}$	Regulatory T cells ($CD4^{-}$)
	$CD3^{+}$ $CD4^{+}$ $CD127^{+}$ $GATA3^{+}$	Th2 cells
	$CD3^{+}$ $CD4^{+}$ $CD127^{+}$ $TBET^{+}$	Th1 cells
	$CD3^{+}$ $CD4^{+}$ $CD127^{+}$ $ROR\gamma T^{+}$	Th17 cell
